# Promoting Green Concepts and Environmental Awareness by a Recycling Expanded Polystyrene with Acetone Experiment and an Interactive Digital Tool

**DOI:** 10.1021/acs.jchemed.5c00800

**Published:** 2026-02-26

**Authors:** Miguel Á. Hernández, Sonsoles Leguey, Alejandro Cortes, Marta Muñoz

**Affiliations:** † Department of Applied Mathematics, Science and Engineering of Materials and Electronic Technology ESCET, 16776 Rey Juan Carlos University, C/Tulipán s/n, 28933 Móstoles, Madrid , Spain; ‡ Department of Financial Economics and Accounting FCEE, 16776 Rey Juan Carlos University, C/Paseo de los Artilleros s/n, 28032 Vicálvaro, Madrid, Spain; § Institute of Research on Technologies for Sustainability (ITPS), 16776 Rey Juan Carlos University, C/Tulipán s/n, 28933 Móstoles, Madrid, Spain

**Keywords:** General Public, Public Understanding/Outreach, Hands-On Learning/Manipulatives, Computer-Based Learning, Green Chemistry

## Abstract

Recycling plastic waste is a major environmental challenge that requires educational initiatives to raise the awareness of and promote sustainable practices. This work demonstrates the educational effectiveness of a hands-on recycling experiment, supported by the interactive digital tool Wooclap, in promoting environmental awareness and introducing green concepts. To achieve this, a hands-on experiment recycling expanded polystyrene (EPS) with acetone was conducted for three age groups: (i) young children, (ii) high school students, and (iii) undergraduate students. Initial and final tests on the Wooclap platform measured the prior knowledge and learning outcomes of the participants. Additionally, participants completed a satisfaction survey to evaluate their satisfaction with the workshop and the knowledge gained. All groups recognized plastic accumulation as a serious environmental issue, but many preuniversity students were unfamiliar with basic green concepts, especially regarding specific types of plastic and their recycling process. These observations highlight the need for educational initiatives like that presented in this work. All groups showed a strong grasp of the concepts related to EPS recycling after the workshop, highlighting the effectiveness of this educational experience. Additionally, participants found the experience enjoyable, which had a positive effect on their learning.

## Graphical abstract

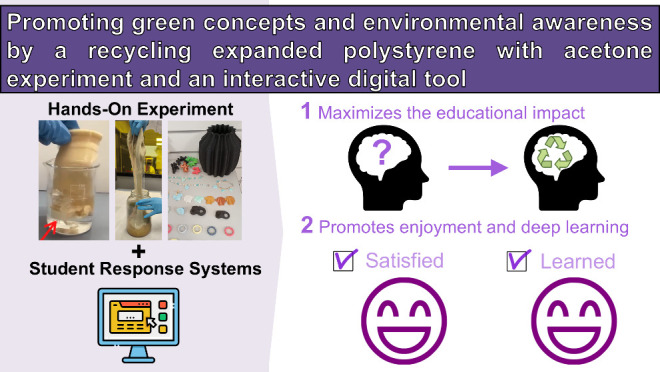

## 1 Introduction

The sharp growth of the world population, the development of industries, and consumer culture have led to a rapid rate of consumption of natural resources and energy, as well as the production of a massive amount of waste.^1,2^ This represents a meaningful problem for environmental sustainability, since waste primarily ends up in landfills, incinerators, or even in oceans, contributing to the depletion of natural resources and the pollution of ecosystems.^3,4^ The transformation of waste into new products through recycling is widely regarded as one of the most efficient approaches to mitigating environmental impact, conserving resources, saving energy, and reducing waste and pollution.^5,6^

The need for recycling is particularly crucial for plastic materials due to their vast production and slow degradation. Global plastic production is extensive and continues to experience exponential growth, because of its remarkable properties, including being lightweight, easy to process, durable, inert, and cost-effective.^7,8^ Furthermore, in contrast to other materials, plastics in a natural environment are highly resistant to degradation, taking hundreds or even thousands of years to fully disappear.^6,9^ Specifically, expanded polystyrene (EPS), also commercially known as styrene, is a plastic particularly harmful to the environment and challenging to recycle. Because of its low cost, lightweight, and excellent insulation properties, EPS is widely used in packing, construction, and food service.^9^ One of its most notable properties is durability; EPS can last several decades with minimal degradation.^10^ However, when EPS is used in short-lived consumer products such as cups, trays, or containers, this stability becomes an environmental drawback, making EPS difficult to eliminate. Additionally, EPS breaks down into microplastics that harm ecosystems and enter the food chain.^11^ Lastly, the low density of EPS, containing approximately 92–98 wt % air, makes its storage and transport remarkably difficult, due to its high volume-to-weight ratio.^12^ As a result of these challenges, many countries have banned EPS in single-use applications.^13^ Thus, effective EPS recycling is crucial for conserving resources and protecting the environment, reducing plastic pollution, and saving materials and energy.

Processes for recycling EPS include mechanical (physical), chemical, thermal, and solvent-based (dissolution) methods.^14,15^ Mechanical recycling typically involves four stages: the material is shredded into flakes, washed to remove contaminants, centrifuged and dried, and finally extruded at around 200 °C. This route is straightforward, well established, and relatively inexpensive compared with alternative methods. However, the recycled polymer often exhibits reduced thermomechanical properties, and the process is limited by the cleanliness and quality of the EPS feedstock.^16,17^ Chemical recycling breaks polymer chains into monomers that can be purified and repolymerized into high-purity polymers. Two main approaches exist: depolymerization and thermochemical conversion. Depolymerization employs catalyzed chemical reactions to decompose EPS into styrene monomers, which can then be purified and reused. Thermochemical recycling relies on pyrolysis, exposing the polymer to a high temperature without oxygen. Chemical methods suit mixed or contaminated EPS and can yield high-purity products (higher via catalysis than pyrolysis), but they are costly, technically challenging, energy-intensive, and generate CO_2_ and other emissions.^18,19^ Thermal recycling does not recover the polymer but instead recovers energy. In this pathway, the polymer is combusted in a controlled incineration process, converting its chemical energy into heat; however, it also generates significant emissions.^20^ In solvent-based recycling, solvents separate and recover EPS without altering its chemical structure. This process involves three main steps: dissolving the polymer in a solvent or solvent mixture at a controlled temperature; separating the solid and liquid phases by filtration; and precipitating or recovering the solid polymer, which could involve an antisolvent, filtration, or drying.^21,22^ Solvent-based recycling is promising because it is simpler, produces polymers with fewer impurities, is cost-effective, and generate relatively low emissions compared with mechanical, chemical, and thermal methods.^21–24^ Currently, solvent-based recycling methods for EPS are under development. One recent approach dissolves EPS in acetone, followed by reshaping and extrusion using 3D printing to produce value-added products.^25,26^ The primary drawback of using acetone is the release of harmful vapors. Although acetone is a low-toxicity volatile organic compound, industrial-scale emissions can still pose environmental concerns. In terms of the climate impact, solvent-based recycling occupies an intermediate position between mechanical and chemical routes. Mechanical recycling, which avoids solvents, complex reactions, and high processing temperatures, exhibits the lowest impact. Chemical approaches such as pyrolysis or catalysis depolymerization demand substantially higher energy inputs.^27,28^ The footprint of solvent-based recycling is more variable, strongly influenced by the energy mix and solvent recovery efficiency.^29^ Consequently, current development in recycling EPS by a solvent-based method is focused on capturing and recovering evaporated solvent for reuse, promoting greater environmental sustainability and circularity in the process.

Recycling necessarily requires the collective awareness and cooperation of the community.^30^ For this reason, educational initiatives that raise awareness of resource depletion, climate change, and the circular economy are fundamental for promoting a recycling mindset and fostering a society encouraged to protect the environment.^31^ Although these efforts must target the entire population because adopting sustainable habits such as recycling is a shared responsibility, they are particularly crucial for young children and university students. This focus is justified since eco-awareness campaigns in early education have demonstrated fostering lifelong sustainable habits and environmental awareness.^32,33^ Moreover, university students represent the next generation responsible for implementing innovative practices in society.^34,35^ With the goal of teaching concepts related to plastic recycling and fostering eco-awareness from young students to university levels, several greener experiments have been developed.^36–40^ For instance, hands-on activities have been designed to simulate key steps of a typical recycling process, including waste collection, plastic separation, and sorting by sink/float tests, polymer processing through compounding and extrusion, and characterization using microscopy and tensile tests.^38^ These works conclude that hands-on experiments effectively convey green chemistry concepts and help students understand the challenges of recycling plastics. Moreover, they suggest that such initiatives positively impact the environment by encouraging recycling practices.

Hands-on experiments involving EPS dissolution in educational contexts have been described previously.^41–46^ These experiments implement the collapse of EPS upon contact with a solvent, differing in the solvent, educational objectives, and subsequent steps. Acetone is the most common solvent, although lower-toxicity alternatives such as d-limonene (a citrus-derived terpene), ethyl acetate, and various essential oils have also been employed; however, compared with acetone, these sustainable solvents are generally more expensive, require longer drying times, and often leave residues.^47–49^ Two main educational objectives are typically associated with these hands-on experiments. First, EPS dissolution serves as a simple chemical demonstration to introduce polymer solubility concepts.^41–43^ Second, dissolution is presented as a potential recycling pathway for EPS waste, emphasizing its relevance to plastic pollution and environmental issues. For example, EPS packing peanuts have been treated with solvents such as acetone, alcohol, and water.^44,45^ Similarly, these experiments can illustrate concepts of polarity and chemical bonding^45^ or develop recycling strategies based on the observed dissolution.^44^ However, they generally focus only on dissolution without addressing how EPS could be transformed into new products. One study extended this approach by fabricating EPS nanofibers through electrospinning waste EPS dissolved in ethyl acetate, reinforced by measurements of surface tension and wetting, and optimization of electrospinning parameters.^46^ Nevertheless, the requirement for electrospinning equipment makes this experiment difficult to implement in most classrooms and is largely inaccessible to younger students.

A plausible approach to engage students in environmental sustainability and teach related green chemical concepts is gamification, defined as “the use of game design in a non-game context”.^50^ Among gamification activities, Student Response Systems (SRS) stand out as an interactive digital tool that allows teachers to create questionnaires with instantaneous feedback for students.^51^ Some proven advantages of SRS include improved student engagement and participation, as well as enhanced understanding and retention of concepts. Additionally, students receive immediate feedback, and teachers gain real-time data for analysis and adjustments.^51,52^ Currently, several platforms for SRS are available, such as Kahoot!, Plickers, Socrative, Quizizz, and Wooclap. Among these, Wooclap stands out for its ease of use, adaptability to online environments, versatility in creating various question types, and ability to provide real-time feedback.^53^

This work combines, for the first time, a hands-on recycling experiment with an SRS interactive digital tool as part of an integrated educational experience to foster environmental awareness and introduce green chemistry concepts to students. The study aims to answer the following questions:

(1) What prior knowledge do learners from different education levels have regarding plastic-related environmental issues, recycling, and, specifically, EPS recycling?
(2) How effective is the learning experience that combines a hands-on recycling experiment with an interactive SRS digital tool in introducing green chemistry concepts and fostering eco-awareness?
(3) How do students perceive the overall activity, in terms of learning outcomes and enjoyment?

The activity was conducted across three educational contexts: young children, high school students, and undergraduate students. It was designed as a multiapproach experience centered on a hands-on experiment in which used EPS were plasticized in acetone and then reshaped using syringes or by hand to create new products without additional equipment. Students collected the EPS data before the sessions. Participants were first introduced to plastic waste issues and the importance of recycling. The experimental procedure was explained with the support of a video to enhance engagement. Knowledge of green chemistry and recycling concepts was assessed through pre- and post-experiment tests using an SRS on the Wooclap platform. The success of the activity was evaluated based on the data collected during the sessions.

## 2 DESIGN AND METHODOLOGY

### 2.1 Educational Context and Participants

The activity described in this work is part of the project “The plastic invasion!”, funded by the Student Observatory at Rey Juan Carlos University (URJC). The Student Observatory is an organization dedicated to analyzing topics of interest related to students, aiming to develop a clear understanding of students’ profiles and needs. Concretely, “The plastic invasion!” project is focused on the problem of excessive plastics use, examining its impact on the university environment, and proposing sustainable solutions. The activity detailed here, also titled “The plastic invasion!”, was designed to raise recycling awareness and promote understanding on the science behind EPS recycling among diverse age groups, ranging from elementary school children to university students. With this purpose in mind, three distinct educational settings were chosen: (1) a science event titled “The European Researchers’ Night” organized by the Community of Madrid (Spain) for young children (YC) in September 2024; (2) a high school science fair at the high school “Arquitecto Ventura Rodríguez” in Boadilla del Monte (Madrid, Spain) for high school students (HS) in June 2024; and (3) a workshop at URJC (Madrid, Spain) for undergraduate students (US) in December 2024. These contexts were selected to engage participants of different ages and backgrounds with the aim of comparing the impact of the project on audiences with varying levels of prior knowledge and maturity.

A total of 94 participants completed the activity. Figure 1 presents histograms showing the percentage distribution of participants by birth year across the three groups. The activity held during “The European Researchers’ Night”, organized by the community of Madrid, involved 24.5% of participants, aged 6–14. At the high school science fair, 47.9% of the participants were aged 10–16. In the university setting, the workshop was attended by 27% of participants aged 18–25. To ensure effective interaction and management, as well as to maintain safety protocols, the activities were conducted in small groups, with 5–7 participants per session for the YC group and 7–14 participants for the HS and US groups.

**Figure 1:**
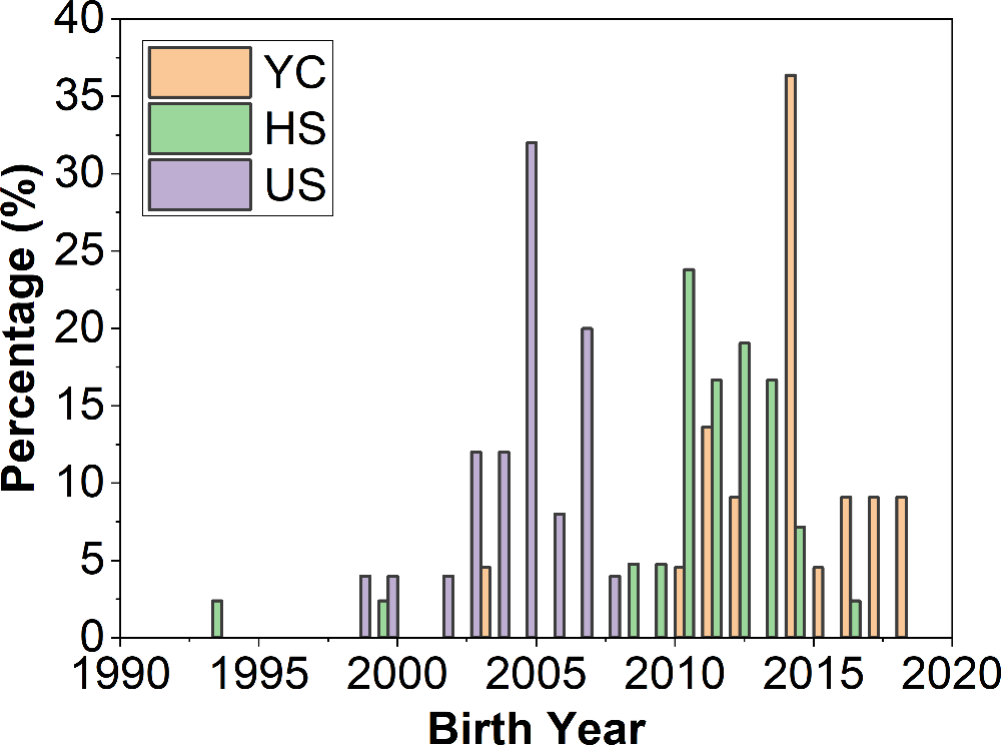
Birth year distribution for the young children (YC), high school students (HS), and undergraduate students (US) groups.

### 2.2 Procedure

The activity consisted of a multipart learning sequence, comprising the following steps:

*(1) Introduction and initial test:* The session began with a brief explanation of the purpose and structure of the experience. Immediately afterward, without any additional information, participant completed an initial test consisting of three parts. The first part was designed to collect demographic information; the responses to the question, “What year were you born?”, are presented in Figure 1. In the second part, participants were asked a yes-or-no question to determine their familiarity with EPS and its recycling methods: “Are you aware of EPS and its recycling methods?”. The third part consisted of a multiple-choice questionnaire assessing the baseline knowledge of the participants about plastics recycling, with each question offering four answer options and only one correct response. An example of a multiple-choice question from the Wooclap initial test is shown in Figure 2. The questions were specifically designed for this study, prepared in advance, and administered in the same format across all age groups. To enhance student motivation and participation,^51,52^ the tests were performed using Wooclap, an interactive digital platform functioning as a SRS. Students accessed the platform by entering a code or scanning a QR code with their personal devices and answered the questions anonymously. There was no limit time for the questions; once all participants had answered, they received immediate feedback on their responses before moving on to the next question.

**Figure 2:**
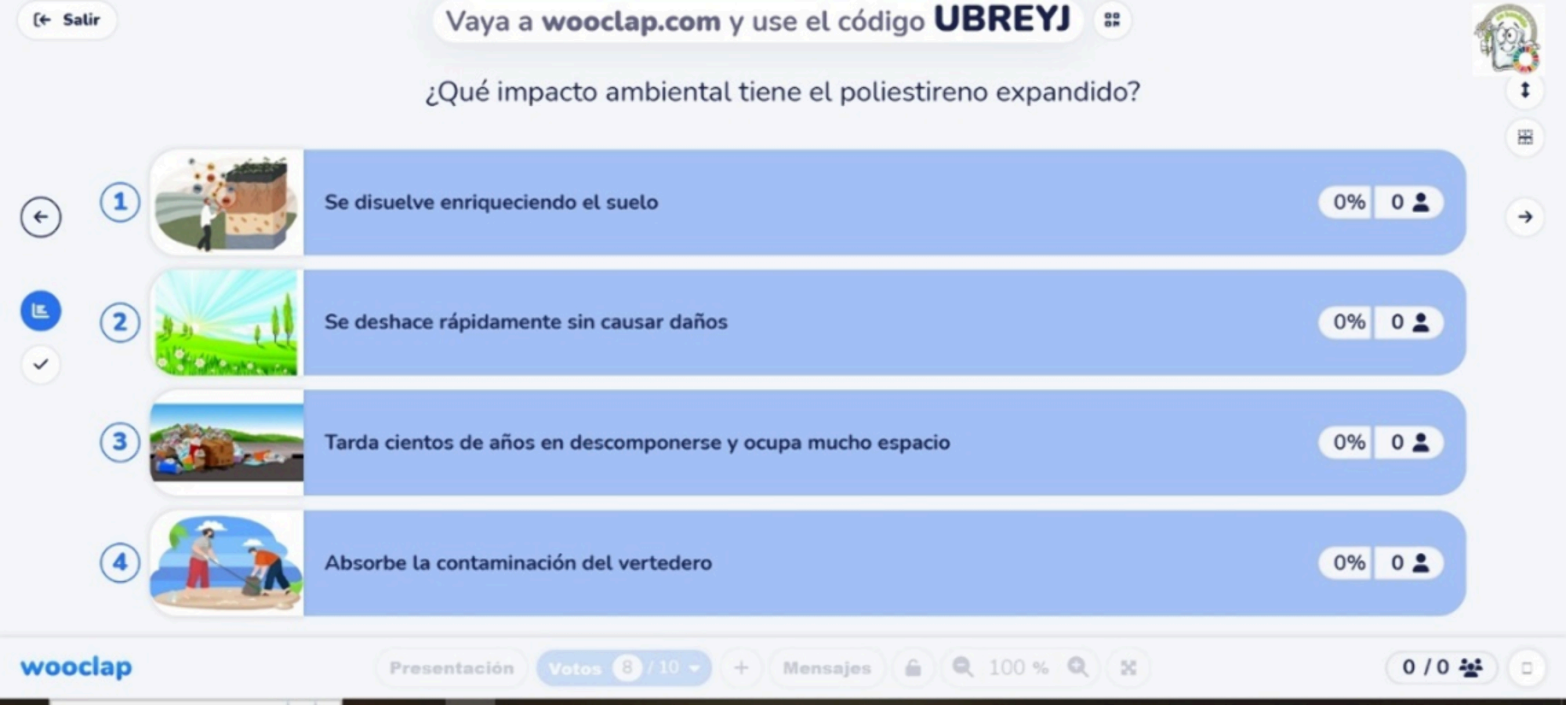
A question from the initial test: What environmental impact does EPS have? The possible answers were: (1) it dissolves, enriching the soil; (2) it breaks down quickly without causing harm; (3) it takes hundreds of years to decompose and occupies a lot of space; and (4) it absorbs landfill contamination.

*(2) Video and explanation of the experimental methodology:* With the purpose of visually introducing the topic and engaging participants, they first watched a 2-min animated video specifically created for this study. Figure 3 provides a schematic illustration of some animations shown in the video. The video opened with the title of the activity “The plastics invasion!”. The screen then filled with EPS trays, followed by the message “Help us stop it”. Next, an open question appeared: “What would happen if our EPS trays were mixed with acetone?” accompanied by animations of a container of acetone and an EPS tray. The following screen displayed the phrase “It gets transformed” along with an animation of a tray dissolving in acetone. The next scene read “Into what you want” while showing a 3D printer creating a flower, symbolizing the shaping of recycling EPS into new parts. The final image represented the phrase “We are waiting for you” together with the activity logo. After the video, the key concepts were reinforced through a detailed explanation covering what EPS is, the environmental impact of EPS waste, the challenges of recycling it, and the use of acetone as an effective solution to recycle EPS. At this stage, participants were encouraged to ask questions, leading to an open-ended discussion. Finally, the methodology for the EPS recycling experiment was explained step by step, as described in the following step.

**Figure 3:**
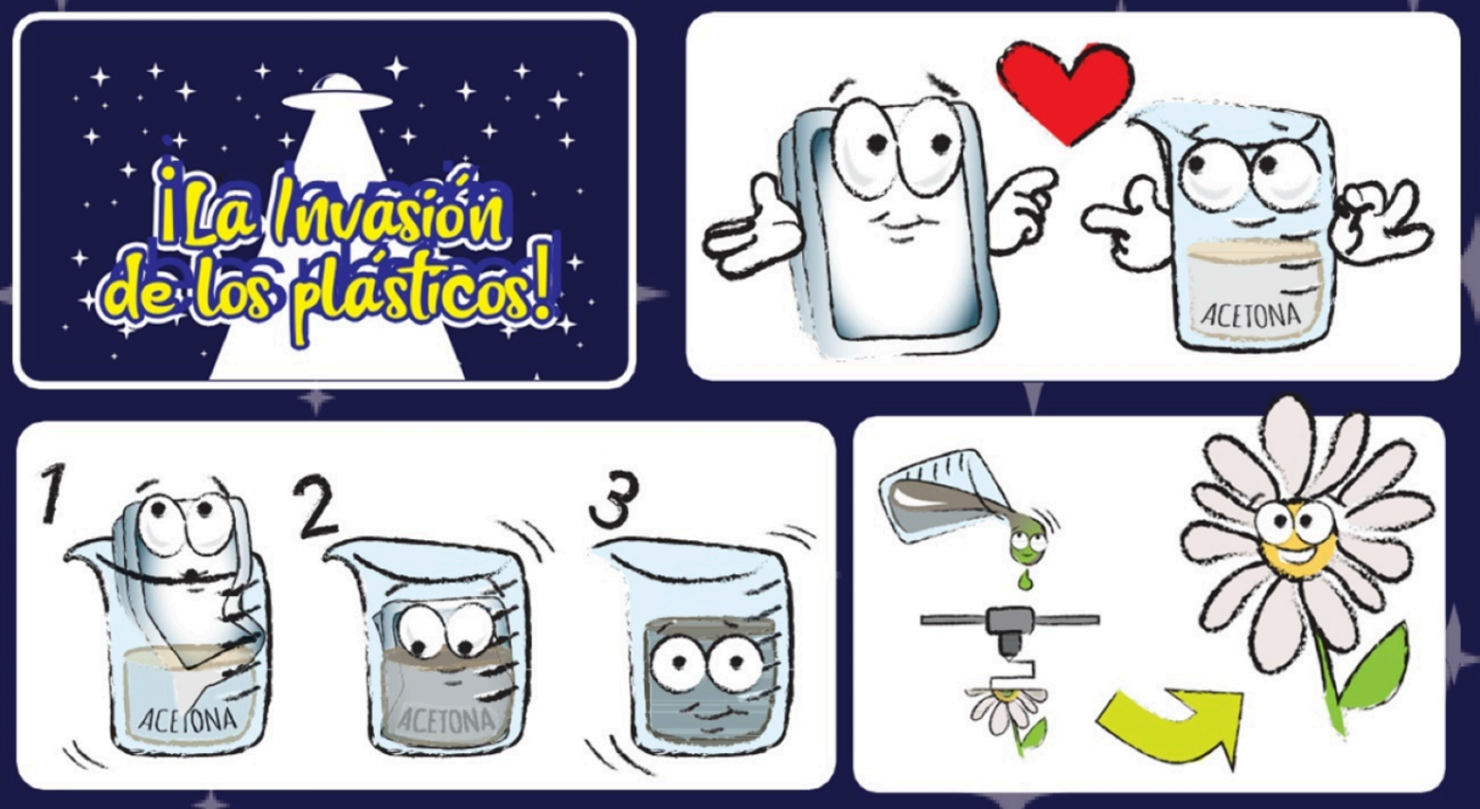
Schematic illustration of the experimental methodology featured in the educational video. It includes the title of the activity, “The plastic invasion!”; the raw materials, EPS trays, and acetone; the process of dissolving EPS in acetone; and the shaping of recycled EPS into new parts.

*(3) Hands-on EPS recycling experiment:* The core of the activity was a hands-on experiment in which participants plasticized used EPS in acetone and subsequently shaped it into new forms. This experiment lasted approximately 30 min. Prior to the workshop, participants were encouraged to collect and bring used EPS. Collection bins, accompanied by an informational poster, were placed in schools, high schools, and universities. Most of the collected EPS consisted of trays, although other containers were also included. To begin, participants broke the EPS into small pieces and placed them in a container with 300 mL of acetone, maintaining an approximate volume ratio of 100:1. During this stage, participants observed how the EPS rapidly collapsed and plasticized when exposed to the solvent. It is important to note that while solvent-based recycling dissolves EPS, in this experiment, the EPS, after being exposed to acetone, collapsed into a solid, moldable mass that could be removed from the solvent, rather than forming a homogeneous solution. Therefore, the EPS was plasticized rather than dissolved. For shaping, disposable 20-mL syringes (labbox) were used to extract the plasticized EPS and inject it into molds (Figure 4a). Alternatively, the plasticized EPS was removed directly and shaped by hand (Figure 4b). Once the acetone evaporated, the EPS hardened completely, retaining the shape given. Previous research has reported that the density of EPS increases from 0.05 ± 0.03 g/mL before treatment to 0.52 ± 0.03 g/mL after solidification. This increase is attributed to the fact that acetone dissolves the polystyrene polymer but not the gas trapped within the foam, which escapes during the process.^26^ This practical experiment provided a direct demonstration of an EPS recycling method, offering participants a tangible example of principles in action. For safety reasons, the students wore lab coats, safety goggles, and nitrile gloves to protect their skin and eyes from accidental splashes of acetone. Furthermore, the experiments were conducted under adult supervision in a well-ventilated area. Although the experiment was safely conducted in a well-ventilated area given the small amounts of acetone used, the rapid dissolution of EPS, and the absence of heating, the use of a fume hood is recommended as best laboratory practice whenever feasible. No unexpected or unusually high safety hazards were encountered in this work.

**Figure 4:**
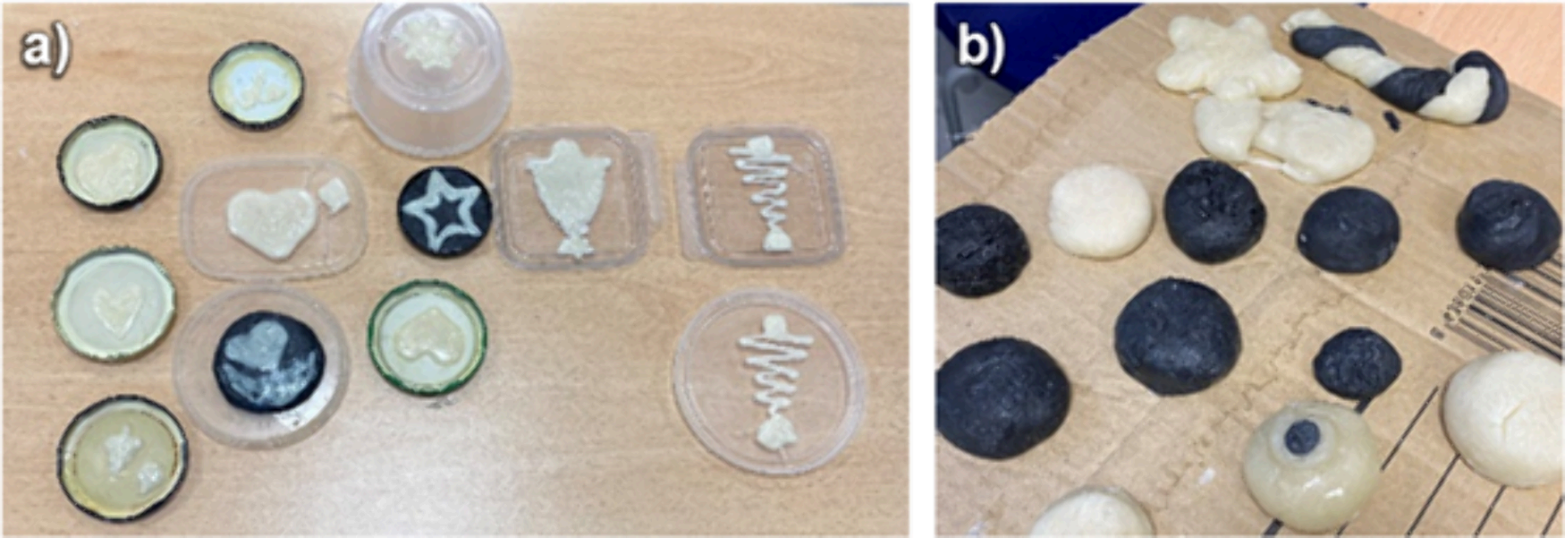
Structures created by the students participating in the workshop using (a) syringes and (b) their hands.

*(4) Final test and satisfaction survey:* The activity concluded with a final Wooclap test to assess the knowledge gained. The final test consisted of multiple-choice questions with four options and a single correct answer. Moreover, the same methodology was applied through the use of the Wooclap platform. Immediate feedback provided to the students helped them identify concepts they had understood and areas that needed reinforcement, thus enhancing the learning experience. The highest-scoring participants were awarded a certificate, which motivated students and encouraged greater participation. Additionally, participant satisfaction was assessed through a survey using a 3-point Likert scale (Very Satisfied–Neutral–Dissatisfied), which gathered feedback on both the overall experience and the perceived level of understanding achieved.

This instructional sequence was designed to accommodate different learning styles, such as hands-on experiments and digital tools, maximizing engagement and comprehension across all of the contexts analyzed.

## 2.3 Data Collection and Analysis

The data collected from this activity comprised demographic information, quantitative measures of knowledge before and after the experience, and survey-based feedback on overall satisfaction and perceived understanding. Participants first completed an initial test to provide demographic details and to assess their baseline knowledge of plastic waste issues, with particular attention to EPS. Following the educational video, hands-on experiment, and explanations, they completed a final test, which was different from the initial one and designed to evaluate the knowledge gained. Additionally, all participants completed a satisfaction survey that captured both their overall satisfaction and their perceived learning outcomes. For both the initial and final assessments, the proportion of correct and incorrect responses was quantified for each question and age group, and responses to the single-answer questions were also summarized. Qualitative observations, including participant engagements, comments, questions raised, and interactions, were also recorded to provide context for the findings.

This study was exempt from review by an ethics committee or Institutional Review Board (IRB), because it was conducted under normal standard education protocols for regular classroom and outreach activities according to the ethical principles and internal regulations of the URJC. Participants voluntarily agreed to take part after being verbally informed about the study and the use of their data. The activities were age-appropriate, noninvasive, and involved no collection of personal or sensitive data or risk to participants. To ensure anonymity, participants used periodic table element names instead of personal identifiers when completing the questionnaires; therefore, no identifiable information was collected.

## 3 Results and Discussion

The percentage of participants who reported being aware of EPS and its recycling methods before the workshop is illustrated in Figure 5. This percentage ranges from 12% for YC to 40% for US. These results indicate that most participants were unfamiliar with EPS and how it is recycled. The US group showed the highest level of awareness, reflecting their higher educational level and maturity. However, overall awareness was low, which is concerning given the widespread use of EPS in daily life. Therefore, despite frequent contact with EPS, most participants were unaware of its name, environmental impact, or how it can be recycled. This observation emphasizes the importance of early education on sustainability and green concepts to promote environmentally responsible behaviors.

**Figure 5:**
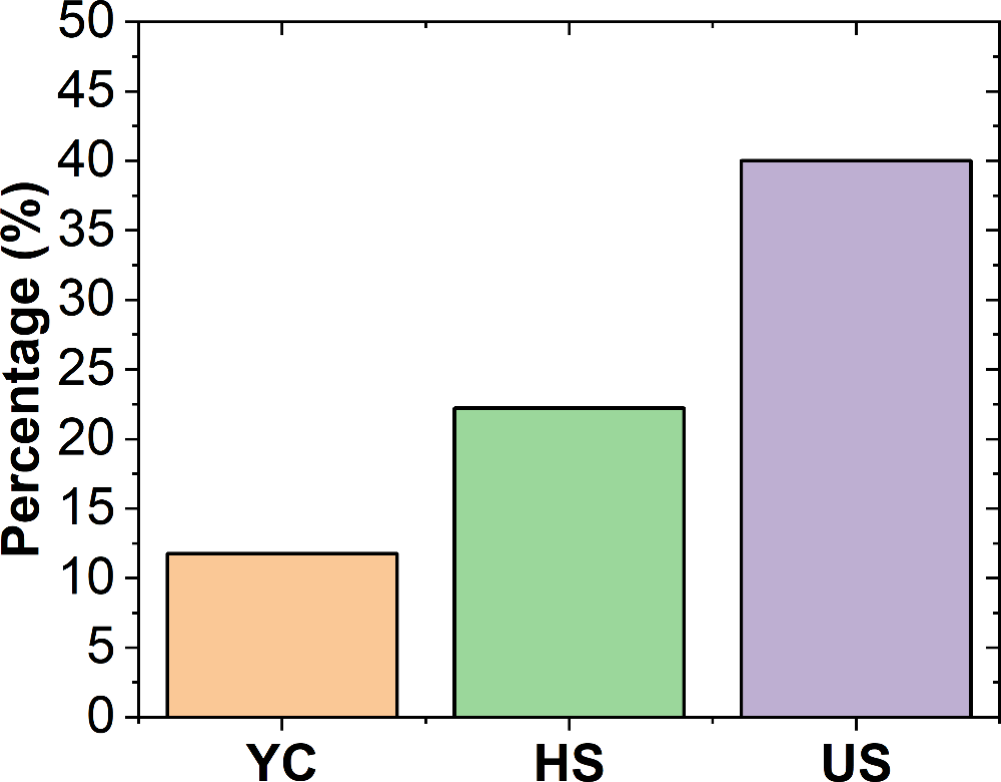
Percentage of participants who were aware of EPS and its recycling methods prior to this activity.

The initial Wooclap test evaluated the baseline knowledge of the participants regarding plastic recycling. Figure 6 presents two representative questions from this test, specifically: “What environmental impact does plastic waste have?” and “Which container is appropriate for disposing of plastic waste?”. The question concerning the environmental impact of plastics received between 86% and 96% correct responses, indicating a general understanding of the problems associated with plastic waste and the importance of recycling. For the question regarding the correct disposal container for plastics, YC and US showed similar results to the environmental impact question. In contrast, the HS group scored significantly lower with only 63% answering correctly. These findings indicate that while most participants recognized the harmful environmental impact of plastic waste and acknowledged the importance of recycling, many (particularly those in the HS group) struggled with practical aspects such as identifying the appropriate recycling bin for plastics.

**Figure 6:**
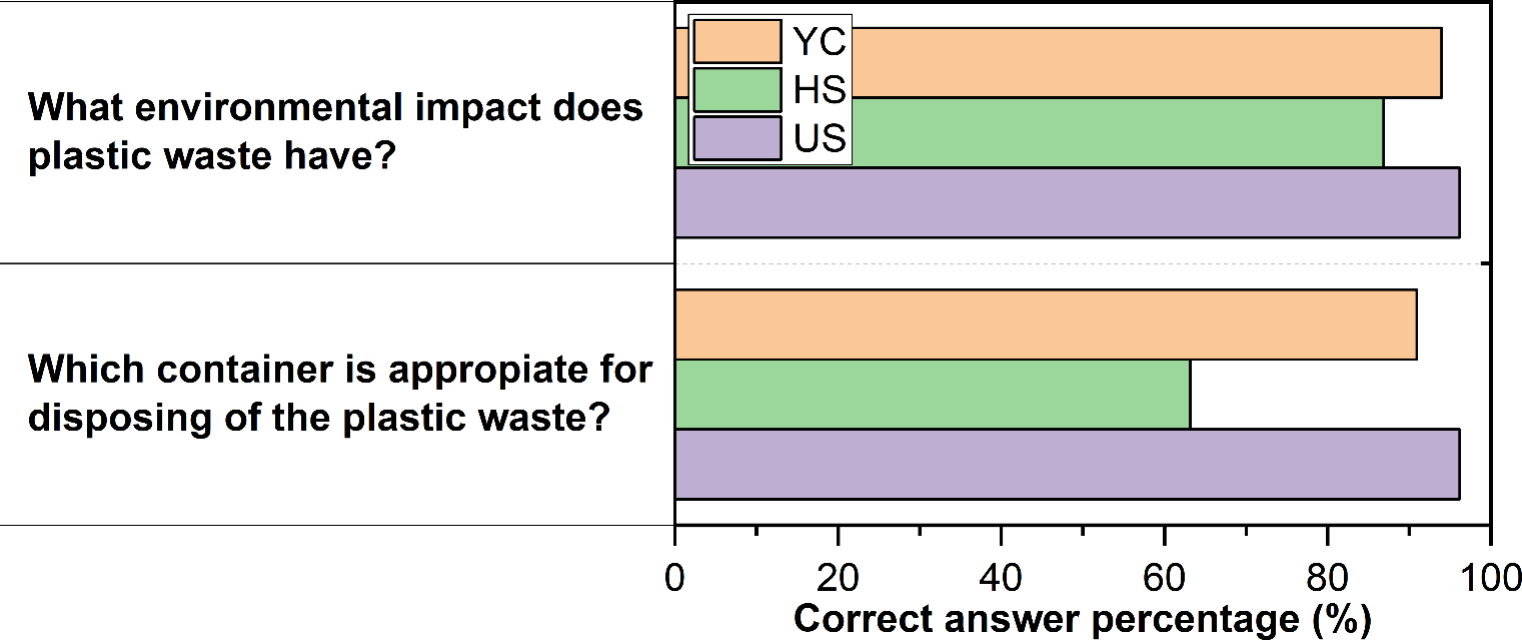
Percentage of correct responses from YC, HS, and US for the questions: “What environmental impact does plastic waste have?” and “Which container is appropriate for disposing of plastic waste?”.

The final Wooclap test, administered after the educational video and EPS recycling experiment with the corresponding explanations, assessed the knowledge gained during the hands-on activity. Figure 7 presents two representative questions from this test, concretely, ″Why is acetone used to recycle EPS?” and “What happens to EPS when it dissolves in acetone?”. The results show a high success rate, with scores ranging from 89% to 100% across all groups, indicating a strong understanding of the concepts following the activity. Considering the initial lack of awareness about EPS and its recycling process (only 12%, 22%, and 40% for YC, HS, and US, respectively, were aware of (Figure 5)) combined with the higher difficulty of the final questions, the observed improvement in comprehension is particularly noteworthy. This enhancement is particularly evident in the YC group: despite 88% being unaware of EPS prior to the activity, they achieved a perfect score (100%) on the final test, matching the performance of the US group. These findings suggest that an educational experience integrating hands-on experimentation with digital tools is an effective strategy for enhancing understanding of the environmental impacts of plastic waste and the recycling of EPS. Previous studies on hands-on activities support these results, showing that direct interaction with materials helps make abstract concepts more tangible, enhancing both understanding and retention.^36–40^ In our study, the hands-on experiment prompted participants to ask more questions about the topic, suggesting that it fostered deeper engagement and potentially enhanced eco-awareness. The use of Wooclap for pre- and post-testing may also have supported focus and motivation, reinforcing learning and retention. While the independent effect of the SRS was not measured in this study, this observation is consistent with prior research reporting that SRS use can increase engagement, participation, and improved learning outcomes.^51,52^ In summary, combining hands-on experiments with SRS tools within a single educational experience is associated with improved learning outcomes, making this a highly effective strategy for teaching sustainability concepts and promoting environmental awareness.

**Figure 7:**
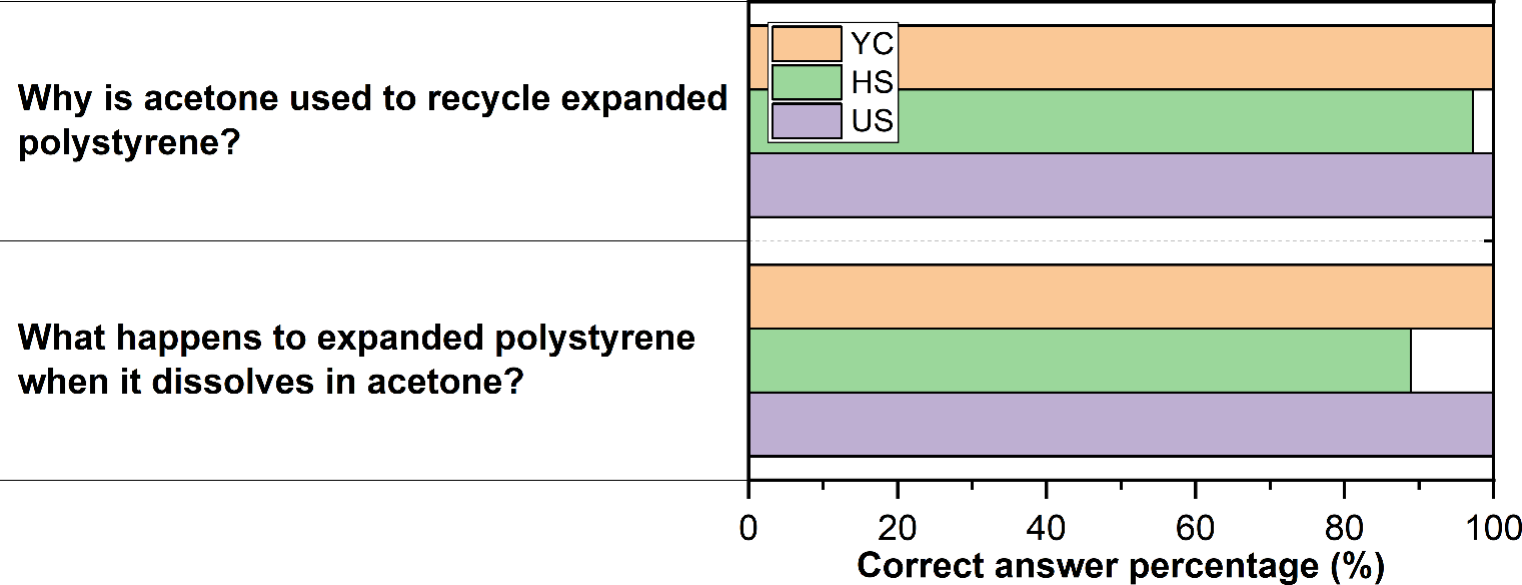
Percentage of correct responses from YC, HS, and US participants for the following questions: “Why is acetone used to recycle EPS?” and “What happens to EPS when it dissolves in acetone?”.

Participants also completed a Likert-scale survey to evaluate both their overall satisfaction with the workshop and their perceived learning outcomes, responding to the following questions: “Are you satisfied with the workshop?” and “Are you satisfied with the knowledge gained?”. Figure 8 shows the percentage of students who reported being satisfied with the workshop and the knowledge gained, as indicated in the survey. It should be noted that participants who were not satisfied with the workshop or the learning outcomes remained neutral and no participants reported being dissatisfied. According to this survey, 100% of participants in the YC and HS groups and 90% in the US group expressed satisfaction with the activity, highlighting strong enjoyment of the workshop, particularly among the YC and HS groups. Additionally, 97%–100% of participants across all groups stated that they had gained valuable insights into EPS recycling. Interestingly, 10% of the US group, although neutral about the workshop itself, reported satisfaction with the learning outcomes. These findings support the idea that combining a hands-on experiment with SRS can be a promising approach to increasing engagement and perceived learning value. This conclusion is further reinforced by the final test results, which showed high levels of understanding across all of the groups. Similar positive feedback on the use of hands-on activities and SRS has been reported in previous studies, which suggest that such methods boost engagement and motivation, thereby increasing the effectiveness of educational activities.^38–40,51,52^

**Figure 8:**
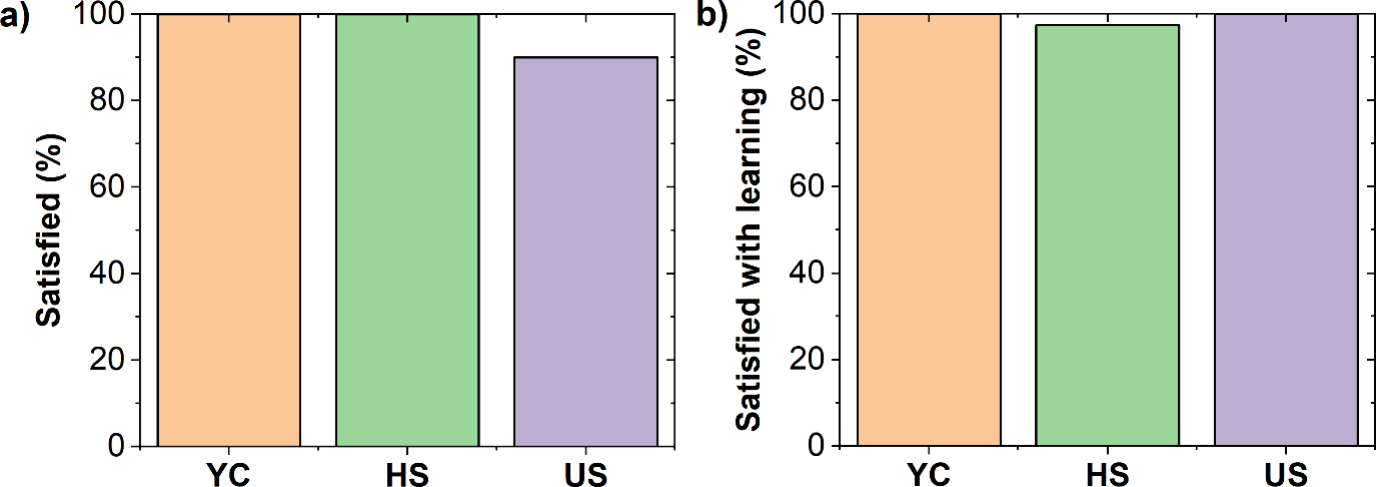
Percentage of participants who reported being satisfied with (a) the workshop and (b) their learning experience across different age groups.

## 4 Conclusions

This work investigates, for the first time, the combined impact of a hands-on recycling experiment and the interactive digital tool Wooclap in designing an educational activity aimed at promoting environmental awareness and introducing sustainability concepts to young children, high school students, and undergraduate students. To achieve this, a multipart learning sequence was developed, including a recycling experiment involving the plasticization of used EPS in acetone and its reshaping into new forms, along with an educational video and associated explanations. Furthermore, pre- and post-experiment tests were conducted through the Wooclap platform to assess the prior knowledge of the participants and the understanding they gained from the activity. The two main conclusions derived from this work are as follows:

(1) Participants demonstrated an initial understanding of the problems associated with plastic waste accumulation. However, many preuniversity students lacked familiarity with everyday green sustainability concepts. This knowledge gap is particularly evident when participants are asked about specific types of plastic and their recycling processes. Most participants were unaware of EPS, the challenges linked to its recycling, and the process involved, despite regularly encountering the material in their daily lives. These findings underscore the need for educational initiatives such as the one implemented here.
(2) The educational experience, combining a hands-on experiment in which EPS is recycled by plasticizing it in acetone with the interactive digital tool Wooclap, appears to have a high educational impact, encouraging deeper reflection, increasing questioning, and a potential strengthening of eco-awareness. It also fostered a more enjoyable and effective learning experience.
